# Multiplex isothermal solid-phase recombinase polymerase amplification for the specific and fast DNA-based detection of three bacterial pathogens

**DOI:** 10.1007/s00604-014-1198-5

**Published:** 2014-02-18

**Authors:** Sebastian Kersting, Valentina Rausch, Frank F. Bier, Markus von Nickisch-Rosenegk

**Affiliations:** 1 Fraunhofer Institute for Biomedical Engineering IBMT, Branch Potsdam, Am Muehlenberg 13, 14476 Potsdam-Golm, Germany; 2 Institute of Biochemistry and Biology, University of Potsdam, Karl-Liebknecht-Str. 24–25, 14476 Potsdam, Germany

**Keywords:** Isothermal amplification, RPA, Microchip, DNA sensor, Point-of-care

## Abstract

We report on the development of an on-chip RPA (recombinase polymerase amplification) with simultaneous multiplex isothermal amplification and detection on a solid surface. The isothermal RPA was applied to amplify specific target sequences from the pathogens *Neisseria gonorrhoeae*, *Salmonella enterica* and methicillin-resistant *Staphylococcus aureus* (MRSA) using genomic DNA. Additionally, a positive plasmid control was established as an internal control. The four targets were amplified simultaneously in a quadruplex reaction. The amplicon is labeled during on-chip RPA by reverse oligonucleotide primers coupled to a fluorophore. Both amplification and spatially resolved signal generation take place on immobilized forward primers bount to expoxy-silanized glass surfaces in a pump-driven hybridization chamber. The combination of microarray technology and sensitive isothermal nucleic acid amplification at 38 °C allows for a multiparameter analysis on a rather small area. The on-chip RPA was characterized in terms of reaction time, sensitivity and inhibitory conditions. A successful enzymatic reaction is completed in <20 min and results in detection limits of 10 colony-forming units for methicillin-resistant *Staphylococcus aureus* and *Salmonella enterica* and 100 colony-forming units for *Neisseria gonorrhoeae*. The results show this method to be useful with respect to point-of-care testing and to enable simplified and miniaturized nucleic acid-based diagnostics.

**Electronic supplementary material:**

The online version of this article (doi:10.1007/s00604-014-1198-5) contains supplementary material, which is available to authorized users.

**Figure:**
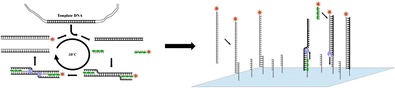
The combination of multiplex isothermal nucleic acid amplification with RPA and spatially-resolved signal generation on specific immobilized oligonucleotides

## Introduction

The amplification of a specific DNA sequence for the detection of a pathogen is a common tool in molecular diagnostics. An established technique for nucleic acid amplification is the polymerase chain reaction (PCR) [1, 2]. However the use of this method in miniaturized lab on-chip devices for point-of-care testing is challenging due to the high electricity demand of the thermocycling process [3]. The design of portable and automated systems for low-resource settings is limited as a result of the high complexity of the reaction setup and the precise temperature control necessary to conduct the amplification.

A possible alternative to the PCR are isothermal nucleic acid amplification techniques which are carried out at a single temperature throughout the entire reaction using different mechanisms e.g. the strand-displacement activity of certain polymerases or the addition of further proteins used in the natural replication processes [4]. Examples for isothermal nucleic acid amplification techniques are the loop mediated amplification (LAMP) [5], the helicase dependent amplification (HDA) [6] the strand-displacement amplification (SDA) [7] and the rolling circle amplification (RCA) [8] which have been recently described and have already proven their use [9, 10]. Another promising method is the recombinase polymerase amplification (RPA) which uses a phage recombinase to direct short oligonucleotide primers to a homologous target sequence. In combination with a strand displacing polymerase and a single-stranded DNA binding protein an amplification of fewer than ten copies of genomic DNA can be accomplished in less than 30 min [11]. In addition the reaction runs at a constant low temperature of about 37–39 °C and the PCR-like system with only two primers offers the possibility for multiplex assays. In the literature some RPA amplifications of different DNA [11–13] and RNA [14–16] targets have been described. So far, few applications of this method have been integrated in more complex instrumentation as an approach for an improved nucleic acid diagnosis in point of care testing [17–21].

In this work, we present the combination of microarray technology and multiplex amplification with RPA. A multiplex RPA reaction was established amplifying different target sequences of *Neisseria gonorrhoeae*, *Salmonella enterica*, methicilin-resistant *Staphylococcus aureus* (MRSA) and of a plasmid reaction control. Although these three pathogens are not related, they do represent important targets for point-of-care testing in the field of sexual-transmitted diseases (STD) (*Neisseria gonorrhoeae*), food borne pathogens (*Salmonella enterica*) and nosocomial infections (MRSA). The use of specific sequences of these three pathogens as targets for the amplification was not to address a clinical relevant question but rather to demonstrate the strength of this method in a highly parallel DNA analysis. Low-density microarrays have already proven their ability as diagnostic tools [22–24] and offer the possibility to test for multiple parameters on a small surface area. Additionally they can serve as detection area in microfluidic chips. The principle of oliognucleotide microarrays is the immobilization of short single stranded nucleic acid probes and the subsequent hybridization of target molecules to these probes. In previous work we demonstrated the combination of microarrays and enzymatic reactions directly on the solid surface, using the probes as template for the enzymes and thus reducing the steps for the diagnostic microarray. PCR [25], RT-PCR [26], the transcription of a whole gene [25] and the isothermal OnChip-HDA [27] on solid surfaces have been described. Solid phase amplification can successfully minimize the formation of primer dimers and thus reduce non-specific products [28] especially during the amplification of multiple targets in parallel where a mixture of different oligonucleotide primers is used.

In our approach the asymmetric RPA is carried out directly on the surface. A labeling of the RPA product is achieved by using a Cy5-fluorophor-modified reverse primer. During the on-chip RPA the amplification in solution as well as the solid phase amplification and hybridization are taking place at the same time in a hybridization chamber with automatic pumps at 38 °C. Successfully amplified products can be detected spatially-resolved by laser scanner or TIRF measurements. To our knowledge this highly multiplex pathogen detection is the first combination of isothermal RPA and microarray technology and offers new possibilities for the development of point-of-care testing devices for nucleic acids.

## Experimental

### Materials and primer design

Oligonucleotide primer and probes used in this study were synthesized by biomers.net (Ulm, Germany, www.biomers.net). For RPA reactions the TwistAmp™ basic kit was purchased from TwistDx (Babraham, UK, www.twistdx.co.uk). Betaine was obtained from Sigma (Schnelldorf, Germany, www.sigmaaldrich.com). Genomic DNA of *Neisseria gonnorhoeae* (DSM9188) was purchased from DSMZ (Braunschweig, Germany, www.dsmz.de). *Salmonella enterica* (DSM14221) and methicillin-resistant *Staphylococcus aureus* (ATCC43300) were extracted using the GeneJET Genomic DNA Purification Kit (ThermoScientific, Waltham, USA, www.thermoscientificbio.com). Quantity and quality of the extracted DNA was determined by measuring A260 and the ratio of A260/A280 on a Nanodrop ND-1000 spectrophotometer (ThermoScientific, Wilmington, USA, www.nanodrop.com). Primers were designed with parameters according to the TwistDx instruction manual and Primer-BLAST available at http://www.ncbi.nlm.nih.gov/tools/primer-blast/ combining Primer3 and BLAST global alignment. Primer dimerization was tested with AutoDimer [29].

### RPA reactions

For the primer evaluation and the optimization of multiplex RPA reactions an extensive testing in solution was carried out before on-chip assays. A typical RPA reaction in a 50 μL volume contained 480 nM RPA primers, 0.5 M betaine and 1x rehydration buffer. This mastermix was used to rehydrate the freeze-dried reaction pellet and 14 mM Magnesium acetate was added to the solution to initiate the reaction. The reaction was kept at 38 °C for 40 min on a thriller thermoshaker-incubater (PEQLAB Biotechnologie GMBH, Erlangen, Germany, www.peqlab.de). Subsequently, amplification products were cleaned-up with the QIAquick PCR Purification Kit (Qiagen, Hilden, Germany, www.qiagen.com) and analyzed on 3 % ethidium-bromide agarose gel with the molecular weight marker HyperLadder V (Bioline GmbH, Luckenwalde, Germany, www.bioline.com) in a BioDocAnalyze imagine system (Analytik Jena AG, Jena, Germany, www.analytik-jena.de). For multiplex experiments primer concentration were adjusted to the following: 360 nM *Neisseria gonnorhoeae*; 340 nM *Salmonella enterica*; 300 nM MRSA and plasmid control. Methicillin-resistant *Staphylococcus aureus* (MRSA) was detected by amplifying a 193 bp fragment of *mecA* which gene product is responsible for the resistance against penicillin-like antibiotics. A 133 bp product of the *invA* gene was amplified in *Salmonella enterica. InvA* is an important virulence factor, necessary for the invasion of cells in the intestinal epithelium and is specific for Salmonella spp. [31]. For *Neisseria gonnorhoeae* the *fit*-gene (fast intracellular trafficker) was chosen as target for the RPA [32], resulting in a 165 bp amplicon. In order to verify a successful amplification of the RPA reaction in a future diagnostic device the amplification of a 227 bp long fragment of a plasmid control was additionally established. All primers used in this study can be found in Table 1.

**Table 1:** Primers used in this study

	Target gene	Oligonucleotide sequence [5′-3′]	Amplicon length
*Staphylococcus aureus* (MRSA)			
SA fwd3	*mecA*	TCCAACATGAAGATGGCTATCGTGTCACAATCGTT	193 bp
SA rev3		CCTGTTTGAGGGTGGATAGCAGTACCTGAGCC	
*Salmonella enterica*			
SE fwd1	*invA*	TACCGGGCATACCATCCAGAGAAAATCGGGCCGC	133 bp
SE rev2		ATTGGCGATAGCCTGGCGGTGGGTTTTGTTGT	
*Neisseria gonorrhoeae*			
NG fwd3	*fit*	CAACGCAATCAAATTCCGTGCGCGAGCCGCAG	165 bp
NG rev2		CCGCGTACGTCTTCCAGCTCAACACCTCCGAT	
Plasmid control			
PC fwd5	Plasmid control	ATTAATGAATCGGCCAACGCGCGGGGAGAGGCGGT	227 bp
PC rev6		CAGCAACGCGGCCTTTTTACGGTTCCTGGCCTT	

For on-chip RPA experiments forward primers were modified with a 5′ NH_2_-(CH_2_)_6_-linker to allow the covalent attachment to the chip surface. Reverse primers in on-chip RPA experiments contain a 5′ Cy5-modification for signal generation

### Preparation of biochips

**Fig. 1:**
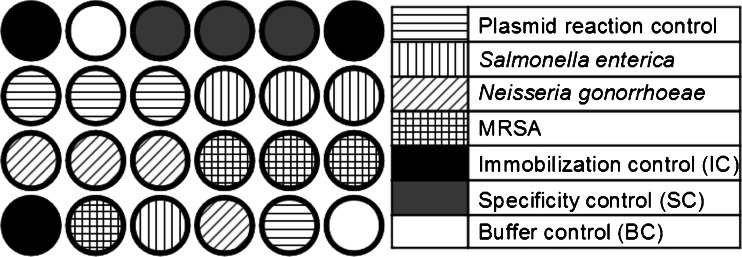
Array layout for on-chip RPA experiments. Each subarray consists of 24 spots. In total nine of these subarrays were spotted on each slide. All subarrays contained target specific oligonucleotides for on-chip amplification and hybridization and different types of controls: Specificity control (SC), buffer control (BC) and immobilization control (IC)

As a solid support, in-house produced epoxy-silanated glass slides were used. Amino-modified oligonucleotide probes were spotted at a concentration of 15 μM in 3xSSC buffer with a Topspot Microarray Printer (BioFluidix GmbH, Freiburg, Germany, www.biofluidix.com). In total nine 6 × 4 subarrays were spotted on each slide (Fig. 1) with an approximate distance of 500 μM between individual spots. Several controls were incorporated in the assay including a specificity control (35 mer oligonucleotide with no sequence homologies), an immobilization control (15mer oligonucleotide with 5′-Cy5-modification) and spotting controls (spotting buffer). The biochips were incubated over night at 25 °C and 75 % air humidity in a climate chamber (Binder, Tuttlingen, Germany, www.binder-world.com). Before on-chip RPA reactions the glass slides were blocked in Thermo Scientific SuperBlock Blocking Buffer (Waltham, USA, www.thermoscientificbio.com) at room temperature for 1 h.

### On-chip RPA

On-chip experiments were performed in a programmable hybridization chamber (Epigenomics, Berlin, Germany, www.epigenomics.com). Each of the chambers has adjustable temperature properties and individually selectable pumps. For an improved hybridization to the immobilized probes an asymmetric RPA was carried out with the reverse primer concentrations being ten times higher than forward primers. RPA reactions on-chip contained Cy5-labeled reverse primers otherwise the concentration of RPA in solution experiments were used. At a constant temperature of 38 °C the reaction was permanently pumped in the reaction chamber with a volume of 8.3 μL at a pump frequency of 2.8 s per stroke for a typical 40 min. After the RPA reaction on the chips and automated washing step was initiated washing the chips three times for 5 min at RT with decreasing concentration of SSC. A wash step with 2xSSC buffer was followed by a second washing step containing 1xSSC and the final wash step comprised of 0.2xSSC buffer. At the end the slides were dried under a stream of nitrogen.

### Read-out and data-analysis

After the on-chip RPA reactions the biochips were read out in an Axon Genepix Professional 4200A microarray scanner (Sunnyvale, CA, USA, www.moleculardevices.com) and data analysis was performed with Genepix 6 software (MDS, Sunnyvale, USA, www.moleculardevices.com). Each signal is defined as the integral of the fluorescence intensity over the whole spot. Contrasts for each fluorescence spot signal were calculated according to the formula:

$$ \mathrm{C}=100\times \frac{\left(\mathrm{S}-\mathrm{Bg}\right)}{\left(\mathrm{S}+\mathrm{Bg}\right)} $$

Where C is the contrast value (in %), S is the primary fluorescence signal and Bg is the average fluorescence signal of the specificity and the negative controls of the individual chip used. In order to verify the results a limit of detection was calculated according to the formula

$$ \mathrm{LOD}=100\times \frac{\left(\left(\mathrm{Bg}+3\times {\mathrm{SD}}_{\mathrm{Bg}}\right)-\mathrm{Bg}\right)}{\left(\left(\mathrm{Bg}+3\times {\mathrm{SD}}_{\mathrm{Bg}}\right)+\mathrm{Bg}\right)} $$

where SD_Bg_ is the standard deviation of the background signal.

## Result and discussion

### Optimization of RPA and multiplex RPA development

**Fig. 2:**
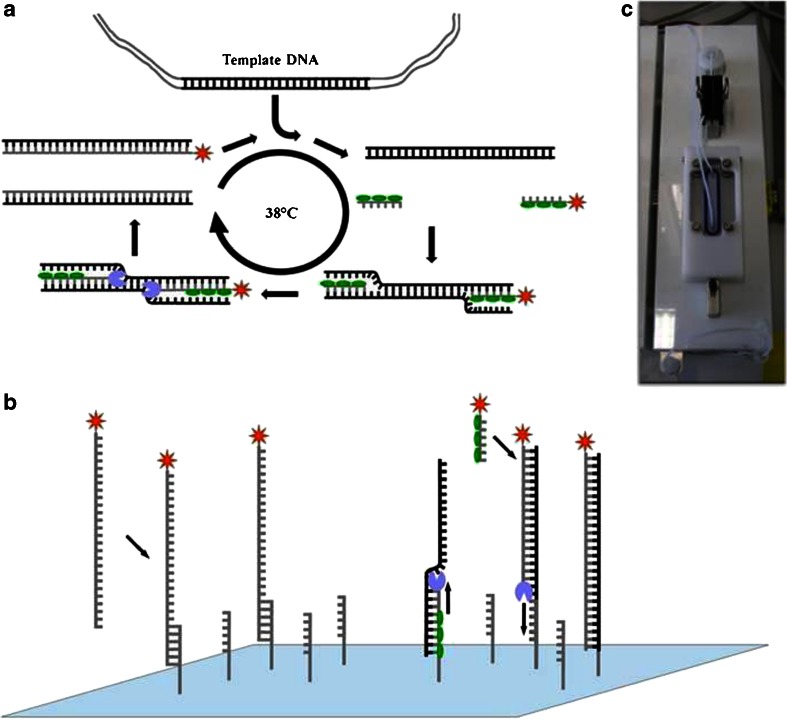
Principle of labeling RPA, on-chip RPA and photograph of one programmable hybridization chamber. **a** Schematic reaction mechanism of the labeling RPA in solution: two oligonucleotide primers form a complex with a recombinase (*green ovals*) and are directed to homologous sequences on the target sequence were they are able to invade the DNA double strand. The polymerase (*blue*) elongates the strand leading to duplication. RPA runs continuously at 38 °C. A labeling of the amplification product is achieved by using a reverse primer coupled with a Cy5 reporter dye (*red*). **b** Two mechanisms for signal generation during the on-chip RPA. (i) Single-stranded and labeled amplicons from asymmetric RPA in solution above the biochip can hybridize specifically to immobilized forward primers. (ii) The forward primer attached to the surface serves as starting point for solid-phase RPA **c** Photograph of one chamber in the programmable hybridization station used for on-chip RPA experiments. The chamber is temperature adjustable and features an individually programmable pump-driven mixing system (not in the picture) with a reaction volume of 45 μL. Slides placed in the chamber are sealed with a silicone o-ring

**Fig. 3:**
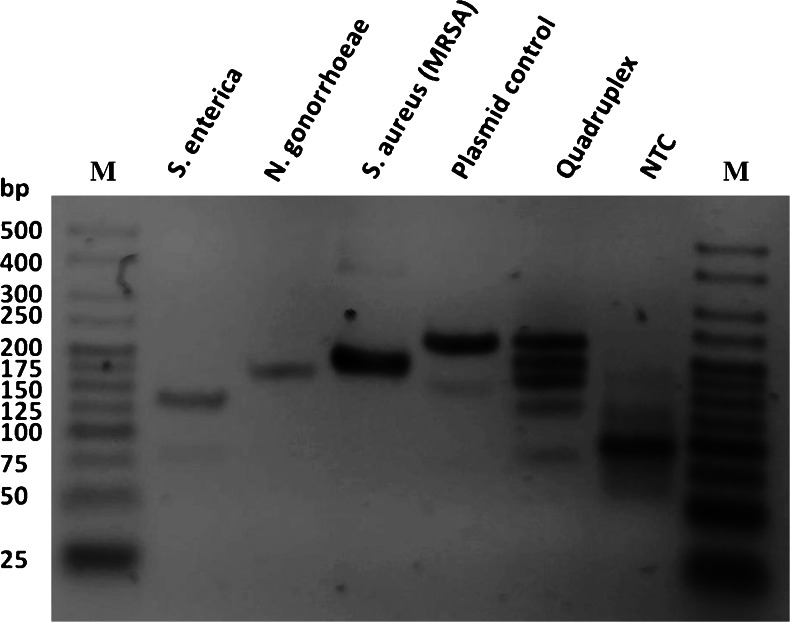
Agarose gel electrophoresis (3 %) with all singleplex and multiplex RPA reactions after clean-up, using 250 pg of genomic or plasmid DNA. Amplified products can be identified by size: *Salmonella enterica* (133 bp) *Neisseria gonnorhoeae* (165 bp), methicillin-resistant *Staphylococcus aureus* (MRSA) (193 bp) and plasmid control (227 bp). A successful quadruplex RPA is indicated by the four distinct bands visible, while in the no-template control (NTC) no specific band is present. M = Molecular weight marker HyperLadder V (Bioline)

In the isothermal RPA a phage derived recombinase in combination with a co-factor form a complex with oligonucleotide primers which then can be directed to homologeous sequences in a DNA template. Subsequently amplification takes place using a strand-displacement polymerase and single-strand binding proteins (SSB) (Fig. 2a). The uniform isothermal reaction at a temperature of 37–39 °C and the use of only two primers are advantageous, yet regular PCR primers are unlikely to work in the RPA [30]. For this reason several primer combinations were initially evaluated for each pathogenic target sequence in singleplex reactions and tested for sensitivity and specificity. Each RPA primer pair generated a product that was distinct in size in order to enable the interpretation of amplification results on an agarose gel. The amplification of multiple DNA targets in the same reaction has the advantage to gain more information from one sample and thus reduce time and costs for every diagnosis since no parallel experiments are necessary to be conducted. So far most isothermal nucleic acid amplification techniques are usually limited to amplify only one target sequence. In the RPA two primers are needed for a successful amplification whereas e.g. the LAMP–technique typically uses six primers. Hence a higher multiplexing ability can be assumed for the RPA. Various primer mixtures and concentrations as well as reaction conditions were evaluated and optimized for the multiplex experiments. In Fig. 3 all simplex and the multiplex detection are visualized on an ethidium-bromide stained agarose gel and the specific amplification with RPA can be determined by the size of the product. All RPA reactions were run at 38 °C for 40 min with 250 pg of genomic DNA and subsequently purified with spin columns. In the quadruplex reaction clearly distinguishable bands of the expected product sizes are observable with no specific band in the no template control (NTC). Albeit the brightness of all bands shows almost the same intensity, minor differences are noticeable. Amplification efficiency in a multiplex amplification assay highly depends e.g. on the target sequence, amplicon size or primer characteristics and has to be optimized for every new multiplex approach.

### On-chip RPA

For point-of-care testing devices the use of agarose gel electrophoresis is not feasible. In the particular case of the RPA, crowding agents and proteins in the reaction can interfere in the run of gels. Therefore amplification products have to be cleaned up before use. This additional step can be very time consuming and requires additional equipment. The on-chip RPA reaction has the benefit that no preliminary preparation steps are necessary before detection. In Fig. 2b the process of the on-chip amplification is described. Herein gene-specific forward primers are covalently immobilized on the solid glass-support using microarray spotting technology. The oligonucleotides are modified with an amino group and bind to the epoxy silanized glass slides. The asymmetric RPA reaction takes place in the solution above the surface inside of an automated hybridization chamber (Fig. 3c). Additionally to the reaction components the solution consists of the Cy5-modified reverse primer and, at a tenfold lower concentration, the forward primer. Our observation shows that an amplification under asymmetric primer conditions is conducive to the reaction and enhances the signal significantly. Especially when only low amounts of target DNA are present. For this reason we conclude that two mechanisms play a role in the signal generation in our on-chip RPA experiments. (i) The excess of reverse primer in the reaction and the subsequent polymerase elongation leads to a preferred amplification of a single strand antisense DNA strand which can hybridize to the target specific probe structure on the surface. During the amplification reaction labeled primers are integrated in the amplicon. The product can then be detected spatially-resolved on the surface. (ii) The immobilized primer can serve as starting point for solid-phase isothermal amplification. A complex is formed between the recombinase and the primer which then can bind to the template DNA and lead to a strand exchange. In the following the polymerase elongates the strand and releases the target DNA which results in an exponential amplification of the DNA molecules. The reverse primer in solution with the fluorophor dye can hybridize to the immobilized strand and also functions as starting point for the polymerase. Both solid-phase and solution-based amplification lead to a signal generation which can be read out (Fig. 3b). In other solid-phase amplification assays with an improved surface-to-volume ratio, for example in reduced volumes or with three dimensional surface structures, the rates of these two mechanisms might be altered and the solid-phase amplification might be preferred. In the Figures S2a-d (Electronic Supplementary Material) the singleplex on-chip RPA reactions using 250 pg of genomic DNA of the three pathogens or 250 pg of plasmid DNA for the control plasmid with a reaction time of 40 min are shown. Only at positions where the specific target sequence is located a fluorescence signal can be observed in the laser scanning images. Other spots on the chip with either different target specific probes, non homologous negative controls or spotting controls show no increased signal. The specificity and the successful amplification were also verified using the quantitative data of the signal intensities to calculate an individual limit of detection (LOD) for every experiment. The resulting analysis displays a good correlation to the qualitative data, showing in all assays signal intensities above the LOD for the specific amplification site while the integrated control spots do not exhibit an enhanced signal. Variations in the LOD are due to differences in background intensity on parts of the slides which increase the standard deviation of the background signal. However all signals generated by the on-chip RPA are highly specific and distinguishable from unspecific background noise.

**Fig. 4:**
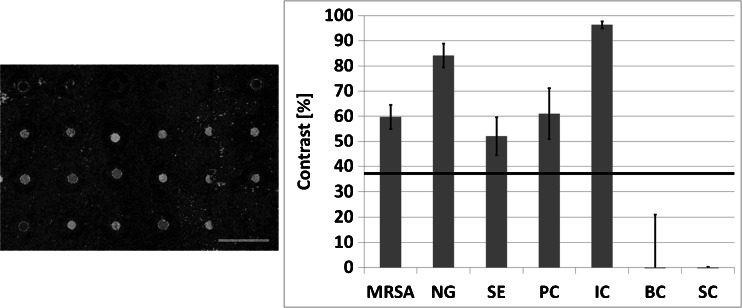
Quadruplex on-chip RPA experiment for the detection of methicilin-resistant *Staphylococcus aureus* (MRSA), *Neisseria gonorrhoeae* (NG), *Salmonella enterica* (SE) and a plasmid (amplification control) (PC) in parallel. Laser scanner images of one subarray (*left side*) showing clearly visible signals at positions where amplification-specific sequences are immobilized (compare with Fig. 1). Quantitative analysis (*right side*) exhibit signals well above the dynamic limit of detection (LOD) (*horizontal line*), while no signals above LOD occur in non-specific or buffer control positions. IC: immobilization control; BC: buffer control; SC: specificity control

In the next step we transferred the multiplex RPA to the on-chip amplification. Herein all four targets were amplified in parallel by mixing all reaction components and the gene-specific primers in the programmable hybridization chamber. The fluorescence image shows a successful amplification of all targets, while no significant signal can be observed at the sites of negative and specificity controls (Fig. 4). Albeit the high background noise which increased the LOD in the quantitative analysis, all amplified targets have a signal above the calculated value while in the negative controls almost no signal can be detected. The signal of *Salmonella enterica* is only narrowly above the LOD which is due to a high signal variance distributed over the biochip. Control experiments with no template show no signal in both qualitative and quantitative analysis (Figure S1e Electronic Supplementary Material). An improved blocking procedure might decrease the signal variation by preventing unspecific binding to the chip surface. The high total amount of fluorophor-labeled reverse primer in multiplex reaction does also increase the background signal. Concentrations of these primers might be reduced, but minimum concentrations for the multiplex experiments without decreasing the amplification efficiency have yet to be defined.

A solid-phase based amplification with immobilized primers has the advantage of potentially reducing non specific product formation for example by primer-dimerization. In our assay a total number of eight oligonucleotides are used in the on-chip reaction. Because of the asymmetric conditions of the assay only low amounts of forward primers remain in the solution while the others are bound to the surface and cannot interfere with the reaction. The attenuation of possible unspecific byproducts is a crucial factor for isothermal nucleic acid amplification methods and in particular for those working at lower temperatures like the RPA where mismatches are more likely to occur. In this study we demonstrated an approach to overcome these problems by solid-phase amplification and space-resolved detection.

### Evaluation of reaction conditions

**Fig. 5:**
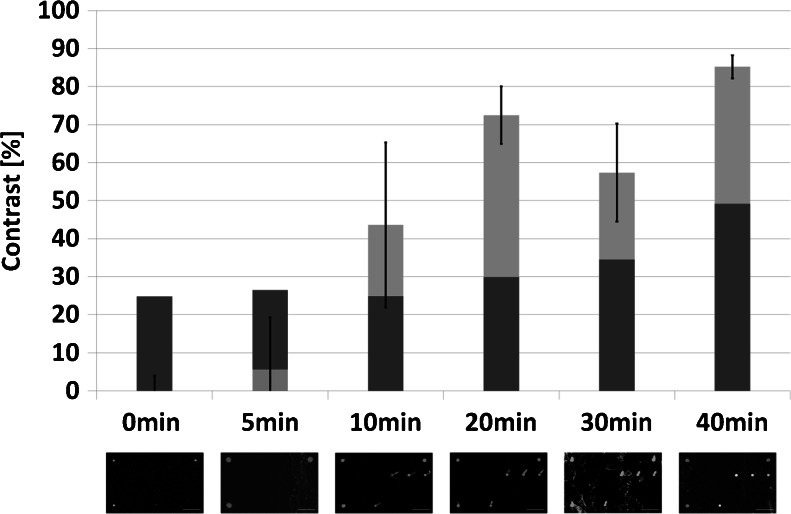
Determination of the reaction time for on-chip RPA experiments showing a signal after 20 min of isothermal amplification for the specific detection of *Salmonella enterica*. After 10 min also target specific signals are visible but are not constant across the whole slide resulting in a high standard derivation. *Dark grey*: Independently calculated dynamic LOD for every assay. *Light grey*: Signal for *Salmonella enterica*-specific positions on slides

The reaction speed for diagnostic test is of crucial importance. The high sensitivity of nucleic acid amplification techniques like the PCR improve the time to result excessively since these methods usually do not require a precultivation of the pathogen. However the PCR still might take hours to be completed when manual prepreparation steps and the analysis procedure are taken into account. In recent years many efforts have been made to improve the speed of the PCR for example with optimized enzymes and highly sophisticated equipment. Yet these instruments are expensive and usually remain laboratory bound. We tested the speed of the on-chip RPA with 1 ng of genomic DNA of *Salmonella enterica* and stopped the reaction at 0, 5, 10, 20 and 30 min by starting the washing process. The results are pictured in Fig. 5 demonstrating the fast isothermal amplification reaction. Stable signals at Salmonella-specific immobilized primers were observed after 20 min reaction time with no unspecific or cross amplification. Even after 10 min positive signals were obtained but in this experiment the signal was not evenly distributed over the entire slide. Therefore a high difference in signal intensities is measured. The only partial mixing of the reaction components in the reaction chamber could be the reason for this occurrence. Although none of the other amplification reactions were tested in the on-chip RPA, the results from the primer optimization and evaluation in solution with real-time quantitative detection indicate that the time ranges are similar for all used primer combinations. A stable amplification reaction can be achieved in less than 20 min. Combined with the subsequent washing step of 15 min and the manual set-up work, the on-chip RPA can be performed within 60 min. At present, external sample preparation steps are time consuming and add up to the total reaction time. A fully automated system could significantly reduce the hands-on time and make the fast on-chip RPA reaction suitable for point-of-care testing where a rapid test result is desired.

The analytical sensitivity of a diagnostic device is an essential criterion. In biological or clinical samples only low amounts of target organisms might be present. Nucleic acid amplification techniques can theoretically detect one single target molecule, or if a target with higher copy number is addressed also one pathogenic organism. RPA has been described in literature to be able to amplify around 10 copies of genomic DNA [11]. We tested the sensitivity of the on-chip RPA with a serial dilution of genomic DNA from methicillin-resistant *Staphylococcus aureus* ranging from 10^6^ to 1 colony forming units (CFU). Each reaction was run for 40 min in the hybridization chamber. We observed an increase in signal intensities in the on-chip RPA with 10 copies of genomic DNA (Figure S2 Electronic Supplementary Material) making it a highly sensitive method for nucleic acid detection. With 100 copies a stable signal well above the calculated LOD is detectable. The amplification of 10^3^ copies shows a clearly positive amplification in the qualitative data of the laser scan. However the quantitative analysis of the data leads to signals just above the LOD resulting from a high, but constant, background signals throughout the slide and thus leading to a relatively low LOD but also equivalent small MRSA-specific contrast values in this assay. Again, an optimization of blocking conditions and washing procedure is necessary to avoid possible misinterpretations and to allow a more constant quantification of the results from on-chip RPA experiments. Similar sensitivities of 10 copies (*S.enterica* and plasmid control) or 100 copies (*N. gonorrhoeae*) can be achieved with other RPA amplification used in this approach.

On-chip RPA reactions were additionally tested in complex samples. For enzyme based nucleic acid amplification techniques methods certain inhibitory substances e.g. hemoglobin, salts or detergents are known. These might be part of a biological sample or can be introduced during the preparation or analysis in the sampling process [33]. The targeted organisms in this assay are usually obtained from nasal (*Staphylococcus aureus*), vaginal or cervical (*Neisseria gonorrhoeae*) swabs, stool, urine, blood or environmental samples (*Salmonella enterica*). All these samples and necessary extraction methods result in specimens containing substances which may block nucleic acid amplification either by inhibiting enzyme activity (directly or by the interaction with cofactors) or forming complexes with the target DNA. Furthermore a high amount of background DNA e.g. human DNA or human cell material in clinical samples might also interfere with the reaction. In order to test a possible influence on the on-chip RPA 1 ng of genomic DNA of *Salmonella enterica* was spiked into 1 μg and 10 μg of salmon sperm DNA, or 1 ng of *Neisseria gonorrhoeae* genomic DNA was spiked into a crude lysate of 2 × 10^4^ Jurkat cells. RPA reactions were run according to the protocol. The results show no significant reduction of signal (Figure S3 Electronic Supplementary Material) in comparison to other on-chip RPA experiments with signals well over the individual determined LODs. These promising results indicate that the RPA might also run in a complex matrix, crude samples or only partially cleaned up specimen. Yet further tests need to determine the inhibitory concentrations for relevant substances for a point of care testing application.

## Conclusion

We described the combination of isothermal nucleic acid amplification with RPA and detection on immobilized probes. The ability to detect multiple targets in the same reaction was successfully demonstrated with the parallel amplification of three different pathogens and a plasmid reaction control. The multiplex amplification was transferred to on-chip RPA assays were the labeled amplification product was analyzed spatially-resolved on a biochip surface manufactured with microarray technology. The on-chip RPA could extend the possibility to detect and analyze nucleic acids in a POC testing device. Without the need for the thermocycling process these devices could be simplified and would not require expensive additional equipment and thus reduce the overall diagnostic costs. Our results show that the on-chip RPA is considerably faster than common PCR techniques. We demonstrated that a time to result in less than 20 min is feasible. At the same time no loss in sensitivity and specificity in comparison with other diagnostic systems can be observed. The use of a solid phase for the detection and amplification allows the parallel analysis of the products on a small surface area and could allow the integration in a microfluidic lab-on-chip device. Yet further optimization of the assay is necessary and the adaptation of the on-chip RPA to a relevant analytical question and the evaluation with clinical samples is considered. By targeting multiple genes at once the on-chip RPA would allow to test e.g. for a panel of infectious pathogens associated with a particular clinical manifestation in a single experiment. In combination with sample preparation and DNA isolation a true field test for nucleic acids could be established with a potential quantitative real-time read-out.

## Electronic supplementary material

Below is the link to the electronic supplementary material.

ESM 1 (PDF 988 kb)
