# Supplementary material for: Multiplex isothermal solid-phase recombinase polymerase amplification for the specific and fast DNA-based detection of three bacterial pathogens
Source: Mikrochim Acta. 2014 Feb 18;181(13):1715–23. doi: 10.1007/s00604-014-1198-5 (PMC4167443; doi:10.1007/s00604-014-1198-5)
Supplement: Supplementary file 1 — (PDF 988 kb) [file 604_2014_1198_MOESM1_ESM.pdf]

Electronic Supplementary Material

Multiplex isothermal solid-phase recombinase polymerase amplification for the specific and fast DNA-based detection of three bacterial pathogens

Sebastian Kersting[1,2], Valentina Rausch[1], Frank F. Bier[1,2] and Markus von Nickisch-Rosenegk[1]

[1] Fraunhofer Institute for Biomedical Engineering IBMT, Branch Potsdam-Golm, Potsdam, Germany

[2] University of Potsdam, Institute of Biochemistry and Biology, Potsdam, Germany

On chip RPA experiments targeting a single pathogen:

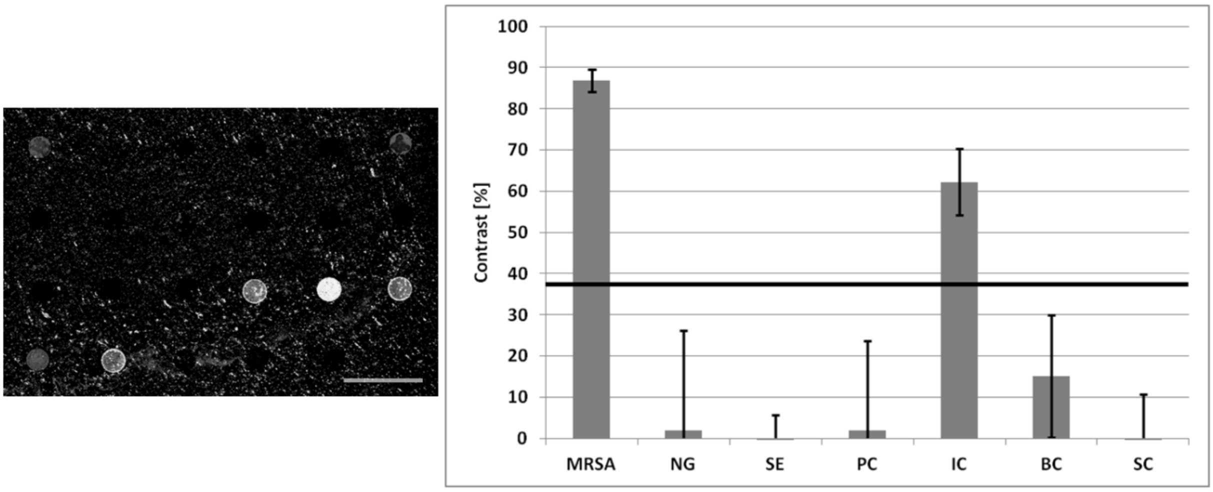

Fig. S1a On chip RPA experiment for the detection of methicilin-resistant *Staphylococcus aureus* (MRSA). Laser scanner images of one subarray (left side) and quantitative analysis (right side); Dynamic limit of detection (LOD) (horizontal line), IC: immobilization control; BC: buffer control; SC: specificity control.

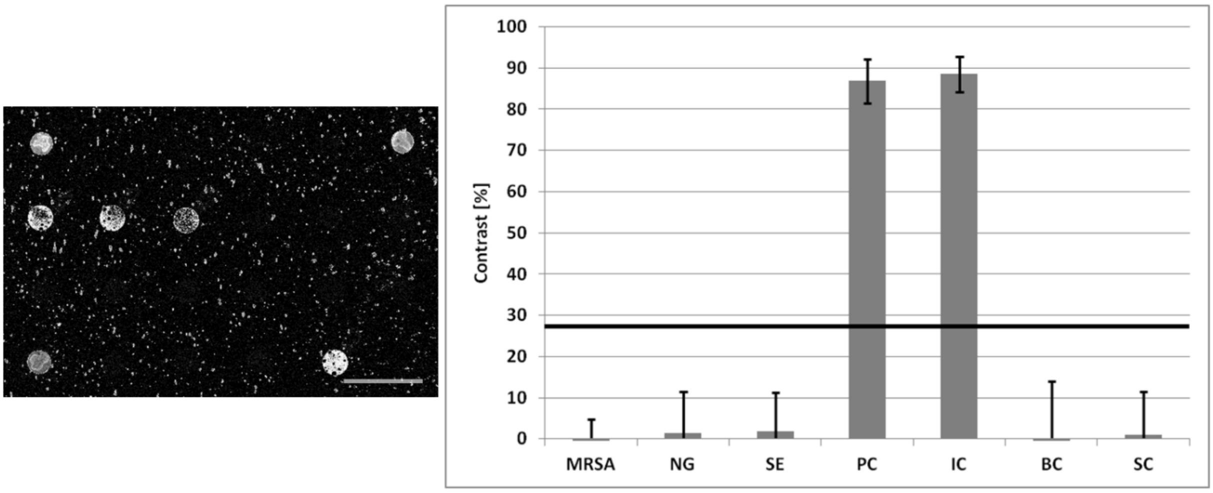

Fig. S1b On chip RPA experiment for the amplification of a plasmid control. Laser scanner images of one subarray (left side) and quantitative analysis (right side); Dynamic limit of detection (LOD) (horizontal line), IC: immobilization control; BC: buffer control; SC: specificity control.

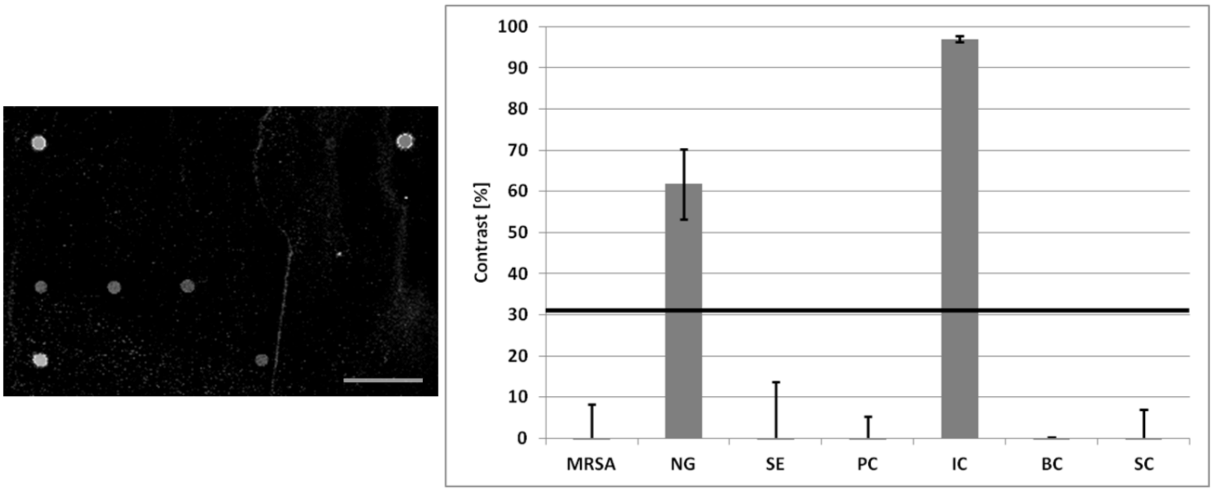

Fig. S1c On chip RPA experiment for the detection of *Neisseria gonorrhoeae* (NG). Laser scanner images of one subarray (left side) and quantitative analysis (right side); Dynamic limit of detection (LOD) (horizontal line), IC: immobilization control; BC: buffer control; SC: specificity control.

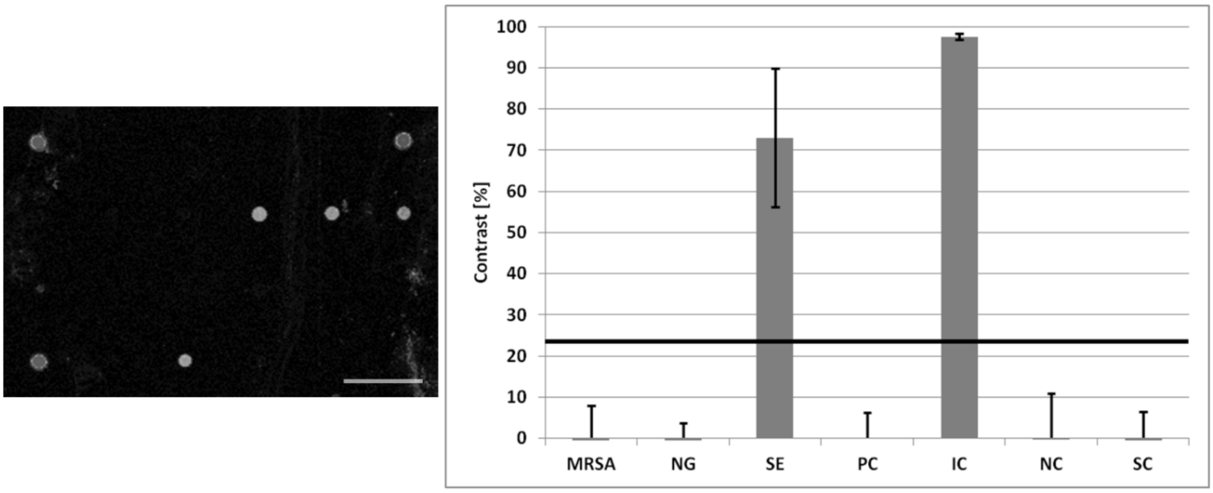

Fig. S1d On chip RPA experiment for the detection of *Salmonella enterica* (SE). Laser scanner images of one subarray (left side) and quantitative analysis (right side); Dynamic limit of detection (LOD) (horizontal line), IC: immobilization control; BC: buffer control; SC: specificity control.

Negative control for multiplex on chip RPA experiments:

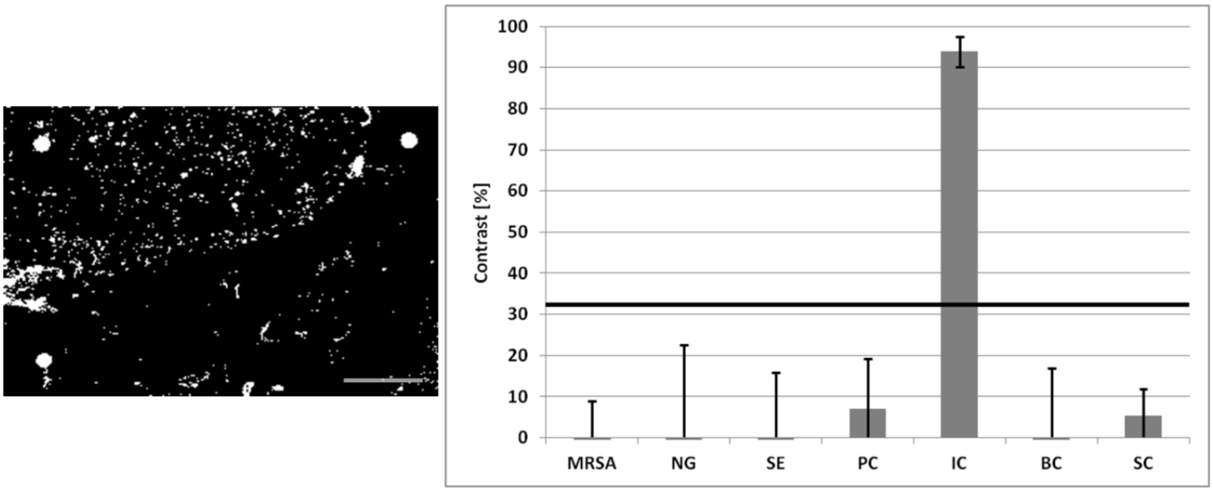

Fig. S1e Control quadruplex on chip RPA experiment using all primer pairs but not template DNAs. Laser scanner images of one subarray (left side) and quantitative analysis (right side); Dynamic limit of detection (LOD) (horizontal line), IC: immobilization control; BC: buffer control; SC: specificity control.

Sensitivity of the on chip RPA:

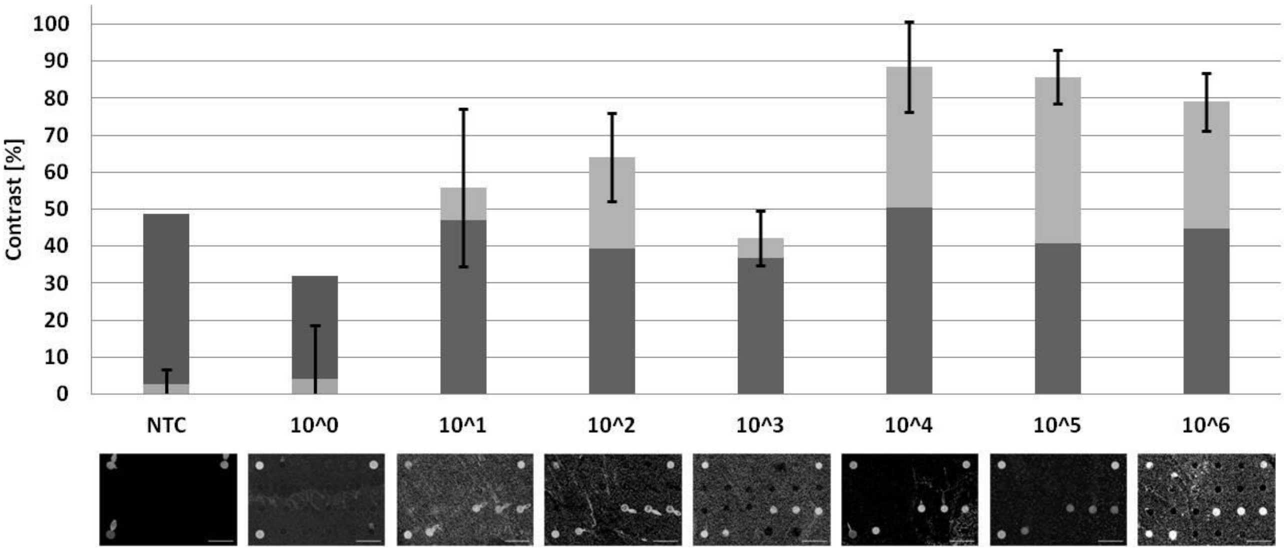

Fig. S2 Sensitivity test for the on chip RPA assay with a serial dilution of genomic DNA from methicillin-resistant *Staphylococcus aureus* from 1 to 10^6 colony forming units (CFU) indicating a successful amplification with 10 copies. NTC: no template control; dark grey columns: dynamic LOD for every assay; light grey columns: Contrast values for MRSA specific signals on slides.

Inhibition experiments:

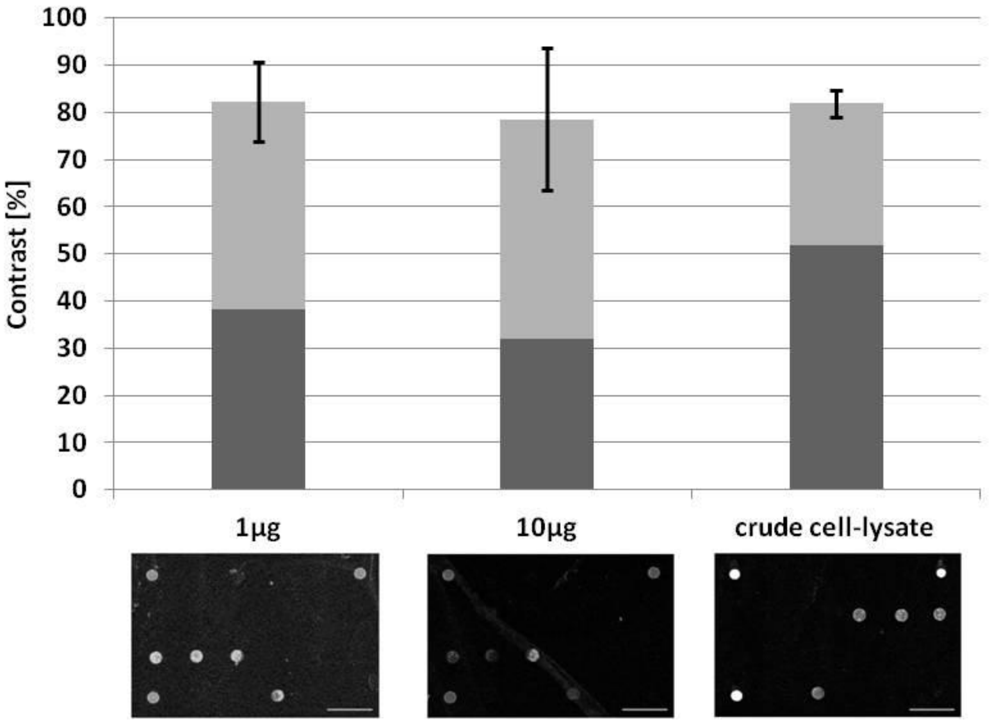

Fig. S3 Inhibition experiments with the on chip RPA show no significant reduction of signal when amplified in complex reaction conditions. 1 ng of gDNA of *Neisseria gonorrhoeae* was spiked into 1 µg and 10 µg of salmon sperm DNA and on chip RPA experiments where conducted according to the previous described procedure. 1 ng of gDNA of *Salmonella enterica* was spiked into a crude cell lysate of $2x10^4$ Jurkat cells and used for on chip RPA without further treatment. Dark grey: Independently calculated dynamic limit of detection for every assay. Light grey: Signal for *Neisseria gonorrhoeae* or *Salmonella enterica*-specific positions on slides.
